# Intratumoral PD-1^high^ CD8^+^ T cells correlate with AFP levels in HCC patients: a brief report

**DOI:** 10.1007/s13402-026-01170-0

**Published:** 2026-02-03

**Authors:** Zuzana Macek Jilkova, Julien Ghelfi, Lucile Dumolard, Christian Sengel, Bleuenn Brusset, Yann Teyssier, Charlotte Costentin, Thomas Decaens

**Affiliations:** 1 Univ. Grenoble Alpes, Institute for Advanced Biosciences, Research Center UGA / Inserm U 1209 / CNRS 5309, Site Santé, Allée des Alpes, La Tronche, Grenoble, 38700 France; 2 Hepato-Gastroenterology and Digestive Oncology Department, CHU Grenoble Alpes , CS 10217, Grenoble, 38043 France; 3 Service de radiologie, CHU Grenoble Alpes, Grenoble, France

**Keywords:** Hepatocellular carcinoma, Alpha-fetoprotein, PD-1, Intra-tumoral PD-1^high^CD8^+^ T cells ratio, Immune checkpoint molecules

## Abstract

**Purpose:**

Hepatocellular carcinoma (HCC), the most common primary liver cancer, typically arises in a context of chronic inflammation driven by metabolic dysfunction, long-term alcohol use, viral hepatitis, and other etiologies. This study aimed to investigate whether intrahepatic and circulating immune profiles in HCC patients correlate with patient characteristics or clinical parameters.

**Methods:**

Fresh tumor tissue, paired non-tumor liver tissue, and peripheral blood samples from 93 patients with HCC were analyzed using multiparametric flow cytometry to characterize lymphocyte subsets (T cells, NK cells, NKT cells, and B cells), immune checkpoint molecule expression (ICOS, 4-1BB, OX40, PD-1, TIM-3, LAG-3, and CTLA-4), and activation status. Associations between immune parameters and patient demographic or clinical features were assessed.

**Results:**

Circulating alpha-fetoprotein (AFP) levels positively correlated with tumor-infiltrating PD-1high CD8+ T cell frequency (r=0.45, p<0.0001), but this correlation was not observed in non-tumoral or circulating compartments.

**Conclusion:**

AFP-producing HCC is linked to intra-tumoral immune exhaustion, marked by PD-1high CD8+ T cell accumulation, suggesting a localized immunosuppressive effect mediated by tumor-secreted AFP.

**Supplementary Information:**

The online version contains supplementary material available at 10.1007/s13402-026-01170-0.

Hepatocellular carcinoma (HCC) represents the majority of primary liver cancers and is one of the leading causes of cancer-related deaths in the world [1, 2]. HCC primarily develops in a chronically inflamed liver, attributable to key risk factors including alcohol consumption, metabolic dysfunction-associated steatotic liver disease (MASLD) or metabolic dysfunction-associated steatohepatitis (MASH), chronic hepatitis B or C virus infection and others. With over 800,000 people diagnosed annually worldwide, HCC is typically characterized by late diagnosis, rapid progression, aggressive invasion, and poor prognosis, all of which make it challenging to treat. Surgical resection, liver transplantation, and ablation are potentially curative treatments for early-stage HCC. Advanced HCC is increasingly treated with immunotherapy, mainly with PD-1/PD-L1 blockade in combination with other drugs targeting immune checkpoint molecules (ICM) or tyrosine kinase inhibitors [2, 3].

Alpha-fetoprotein (AFP) is a 70-kDa oncofetal glycoprotein normally produced by the fetal liver and yolk sac during early gestation [4, 5]. Its serum levels decline sharply after birth and are typically negligible in adults. However, over 50% of HCC patients exhibit elevated AFP [6–8], which differs from fetal AFP in glycosylation patterns [5, 9]. Since its discovery in the 1970s, AFP has been widely used as a diagnostic and prognostic biomarker for HCC, reflecting tumor differentiation, aggressiveness, and invasive potential [4]. High AFP–secreting tumors often comprise poorly differentiated cells with stem-like and progenitor-like features, including rapid proliferation, contributing to an aggressive phenotype [10, 11]. AFP has been correlated with tumor progression, metastasis, and potentially immune evasion [9]. AFP exerts complex immunomodulatory effects depending on its concentration, source, and localization. Circulating AFP can induce apoptosis in immune cells, whereas cytoplasmic AFP inhibits apoptosis in tumor cells [5, 9]. In vitro, AFP upregulation enhances PD-L1 and B7-H4 expression via NF-κB activation in HCC cells, supporting a role in tumor immune escape [12]. AFP receptors (AFPR) are expressed on tumor-infiltrating lymphocytes, suggesting a paracrine AFP/AFPR axis modulating the local immune microenvironment [13, 14].

CD8^+^ T cells are critical for anti-tumor immunity but are frequently exhausted in HCC, exhibiting high levels of inhibitory checkpoint molecules (PD-1, LAG-3, TIM-3), particularly in advanced disease [15]. The functional impairment of these T cells can be partially reversed by checkpoint blockade therapies.

In this study we collected samples from 93 HCC patients, of whom 79 patients were male (84.9%), the median age was 69 [IQR: 63–86] and the median AFP circulating level was 10.56 ng/ml [IQR: 4.3–137]. Fresh liver biopsies (tumor and non-tumor) and whole blood samples were stained for multiparametric flow cytometry using two panels for the identification of the major lymphocyte populations and the expression of ICM.

Frequencies of CD8^+^ T cells, NK cells, and NKT cells were enriched in liver tissue compared to blood (Fig. 1A). The median CD4/CD8 ratio was 1.48 [IQR: 0.91–2.59] in tumoral tissue, 0.98 [IQR: 0.63–1.48] in non-tumoral and 2.96 [IQR: 1.91–4.57] in blood (SI Fig. 1A).

CD69^+^ T cells were significantly increased in tumor (51.0%) and non-tumor liver (48.9%) versus blood (2.4%, *p* < 0.0001), (Fig. 1B), with higher frequencies of CD69^+^ CD8^+^ T cells compared to CD4^+^ T cells, (SI Fig. 1B-C). T cells in liver tissue also exhibited higher ICM expression than in circulation (Fig. 1C-D), with tumor-infiltrating T cells expressing ICOS, 4-1BB, and OX40 more than T cells in non-tumor tissue (Fig. 1C, SI Figs. 1D–E). NK and NKT cells similarly displayed elevated ICM expression intrahepatically (SI Table 1).

Correlation analysis between circulating AFP and immunophenotyping data, focused on all ICM, revealed no significant associations for non-tumor tissue or blood. However, tumor-infiltrating lymphocyte parameters were among the top variables correlated with AFP, with only two remaining significant after p-value adjustment: frequency of intra-tumoral PD-1^high^ CD8^+^ T cells and number of nodules (Fig. 1E). AFP levels positively correlated with PD-1^high^ CD8^+^ T cell frequency (*r* = 0.45, *p* < 0.0001) in the tumor, and these cells were mainly increased in patients with AFP ≥ 10 ng/mL (Fig. 1F). This association was independent on the liver disease etiology, SI Fig. 2.

**Fig. 1:**
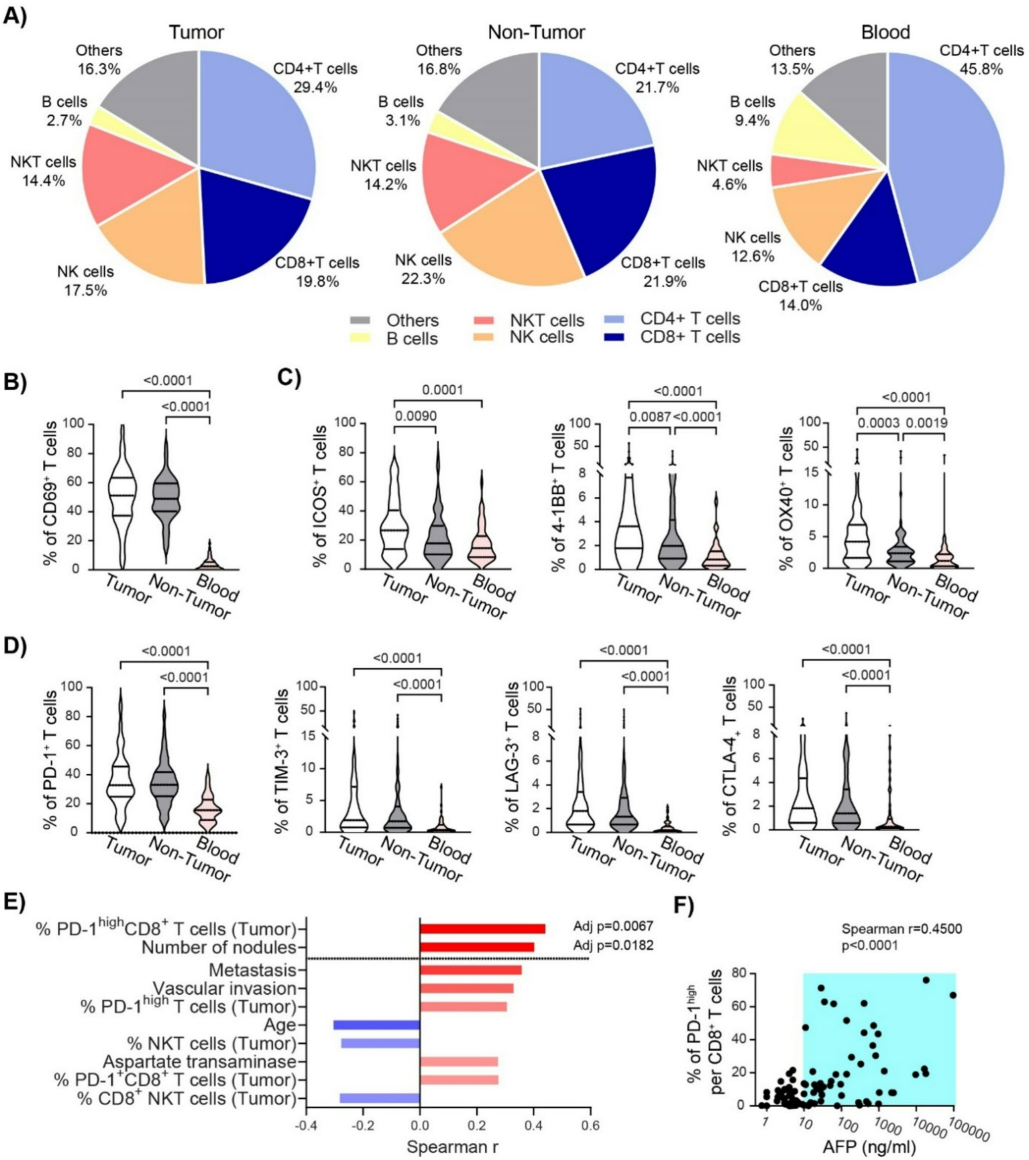
Immunophenotyping of HCC patients and correlation with AFP levels. **A**) Frequency of immune cell populations gated from CD45^+^ lymphocytes in Tumor, Non-Tumor and Blood samples. We identified CD3^−^CD56^−^CD19^+^ B cells, CD3^+^CD56^+^ NKT, CD3^−^CD56^+^ NK cells, and CD3^+^CD56^−^ classical CD4^+^ and CD8^+^ T cells. **B**) The frequency of activated CD69^+^ cells per T cell population. **C**) The frequency of ICOS, 4-1BB and OX40^+^ cells per T cell population in Tumor, Non-Tumor and Blood samples. **D**) The frequency of PD-1^+^, TIM-3^+^, LAG-3^+^ and CTLA-4^+^ cells per T cell population in Tumor, Non-Tumor and Blood samples. Groups were compared by Kruskal-Wallis test. **E**) The top ten list of Spearman coefficients of significant correlations between circulating levels of AFP and clinical, biological and immunological variables. **F**) Correlation between intra-tumoral PD-1^high^CD8^+^ T cell frequency and AFP circulating levels in HCC patients. Each dot represents a patient, (*n* = 93)

Cohort stratification by AFP levels (AFP < 10 ng/mL, *n* = 43; AFP ≥ 10 ng/mL, *n* = 50), determined based on cohort median and prior studies [6, 7], showed that AFP-high patients (AFP ≥ 10 ng/mL) had comparable demographic and clinical characteristics to AFP-low patients (AFP < 10 ng/mL), with a trend toward multiple tumors and more advanced BCLC stage (Table 1). Immunophenotyping showed significantly elevated frequencies of tumoral PD-1^+^CD4^+^ and CD8^+^ T cells in AFP-high patients, with the most pronounced difference in PD-1^high^CD8^+^T cells (21.0 ± 3.0% vs. 5.3 ± 0.8%, *p* < 0.0001; Fig. 2A–B, SI Fig. 3). PD-1^+^ cell counts per mg tissue were also higher in AFP-high tumors (Fig. 2C-D). A substantial proportion of tumor-infiltrating PD-1⁺CD4⁺ T cells may correspond to regulatory T cells (Tregs), as previous study has demonstrated Tregs accumulation within HCC tumors, with higher intratumoral Treg frequencies observed in patients with elevated AFP levels [16]. Frequencies of CD8⁺ T cells expressing other inhibitory ICM tended to be higher in our cohort of patients with high AFP levels compared to AFP-low patients, with the strongest trend observed for TIM-3 expression (SI Table 2). Finaly, we confirmed the immunosuppressive effect of AFP by decreased production of IFN-γ by tumor-infiltrating CD8^+^ T cells ex vivo (Figure S4).

**Fig. 2:**
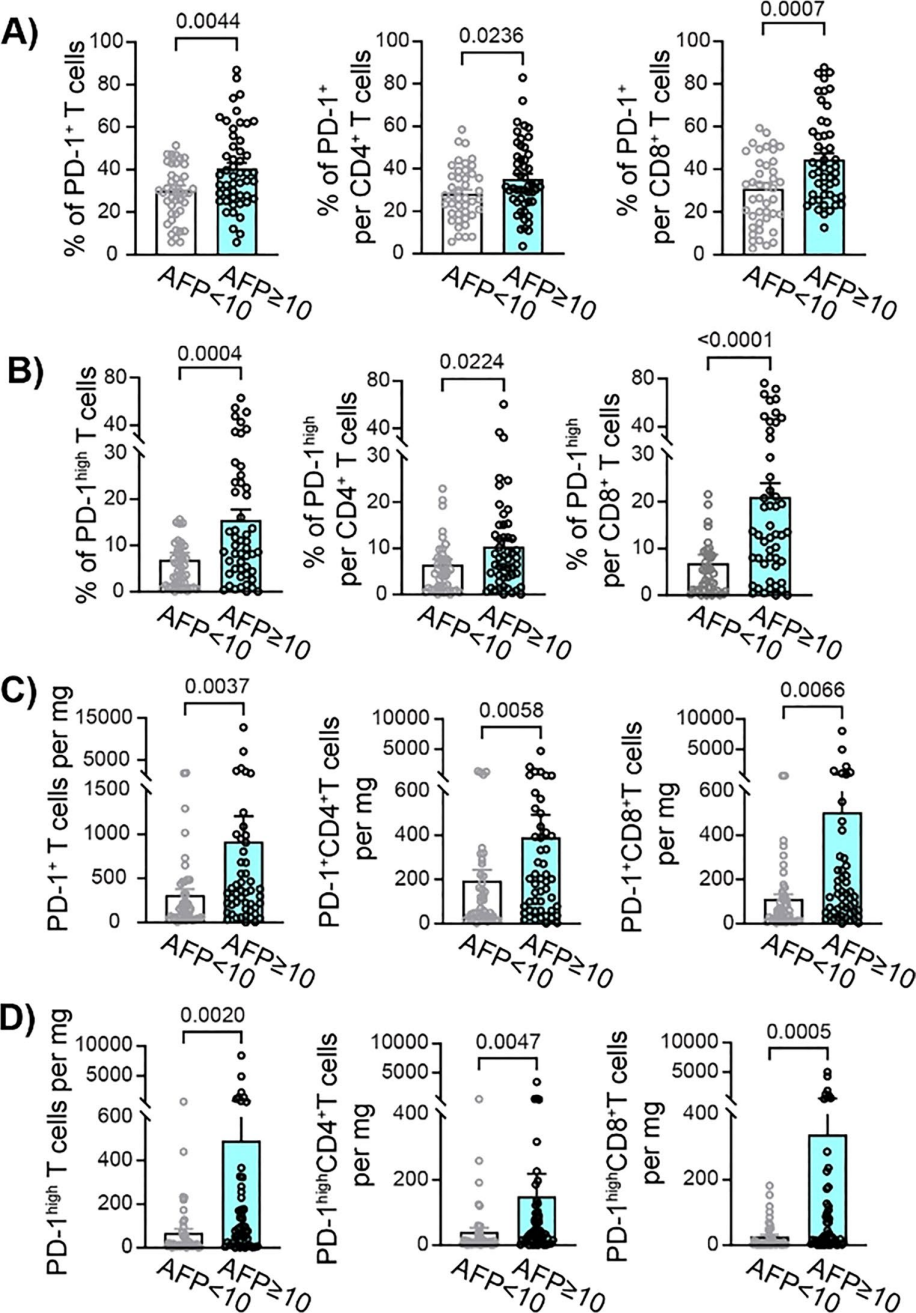
Intra-tumoral PD-1 expression of HCC patients divided based on AFP levels. **A**) Frequency of PD-1^+^ T cells in the tumor of HCC patients with circulating AFP levels lower than 10 ng/ml (AFP < 10, *n* = 43) compared to tumor of HCC patients with circulating AFP levels equal or higher than 10 ng/ml (AFP ≥ 10, *n* = 50). **B**) Frequency of PD-1^high^ T cells in the tumor. **C**) Number of PD-1^+^ T cells per 1 mg of tumor tissue. **D**) Number of PD-1^high^ T cells per 1 mg of tumor tissue. Mann-Whitney U test was used to compare AFP < 10 and AFP ≥ 10 group of patients. Each dot represents a patient; mean ± SE, 2-tailed P value

**Table 1:** HCC patients’ characteristics

Variables	AFP < 10 (*n* = 43)	AFP ≥ 10 (*n* = 50)	*p* value
AFP (ng/mL), median [IQR]	4.0 [2.8–5.1]	97 [26–744]	*< 0.0001*
Age (years), median [IQR]	70.0 [65.0–78.0]	68.0 [62.0-73.3]	ns
Sex, n (%)			
Male	35 (81.4)	44 (88.0)	ns
Female	8 (18.6)	6 (12.0)	
Etiology, n (%)			
Alcohol	11 (25.6)	17 (34.0)	ns
MASLD/MASH	18 (41.9)	20 (40.0)	
Viral	7 (16.3)	10 (20.0)	
History of HCC treatment, n (%)	10 (23.3)	21 (42.0)	ns
Fibrosis stage Metavir, median [IQR]	3.5 [2–4]	4.0 [3–4]	ns
BMI, median [IQR]	27.3 [23.9–30.0]	25.3 [24.0-30.2]	ns
Albumin (g/dL), median [IQR]	3.7 [3.4–4.1]	3.7 [3.2–4.1]	ns
Bilirubin (mg/dL), median [IQR]	0.8 [0.5–1.3]	0.9 [0.7–1.5]	ns
Creatinin (mg/dL), median [IQR]	0.76 [0.66–0.96]	0.75 [0.66–0.96]	ns
PT (%), median [IQR]	85.0 [78.0–95.0]	77.5 [66.8–91.0]	ns
AST (U/L), median [IQR]	39.0 [31.0-63.3]	57.0 [36.0-114.0]	ns
ALT (U/L), median [IQR]	35.0 [27.8–50.0]	42.0 [24.5–251.0]	ns
ALP (U/L), median [IQR]	102 [91–127]	140 [89–981]	ns
GGT (U/L), median [IQR]	115 [61–340]	177 [67–340]	ns
Child Pugh Score, n (%)			
A	39 (90.7)	42 (84.0)	ns
B	4 (9.3)	8 (16.0)	
Tumor size (mm), median [IQR]	33.0 [20.0–48.0]	31.5 [21.8–57.5]	ns
Number of nodules, median [min-max]	1.0 [1.0–4.0]	2.0 [1.0–4.0]	ns
BCLC staging, n (%)			
0	8 (18.6)	3 (6.0)	ns
A	19 (44.2)	16 (32.0)	
B	10 (23.3)	10 (20.0)	
C	6 (14.0)	16 (32.0)	
D	0	5 (10.0)	

HCC: hepatocellular carcinoma; AFP: alpha fetoprotein; n: number; IQR: interquartile range; MASLD: metabolic dysfunction-associated steatotic liver disease; MASH: metabolic dysfunction-associated steatohepatitis; BMI: body mass index; PT: prothrombin time; AST: Aspartate transaminase; ALT: Alanine transaminase; ALP: Alkaline Phosphatase; GGT: Gamma-glutamyl transferase; BCLC: Barcelona clinic liver cancer, ns: Not significant

Our findings reveal that AFP-producing HCC tumors are specifically associated with increased PD-1^high^ CD8^+^ T cells. These results corroborate previous small-cohort studies and recent reports highlighting suppressive immune microenvironments in AFP-positive HCC [17–19]. Notably, only intratumoral, not circulating or non-tumoral, PD-1^high^CD8^+^ T cells correlated with AFP levels, underscoring a localized immunosuppressive effect, suggesting that tumor-secreted AFP directly contribute to tumor growth through modulating immune response.

While elevated AFP is generally associated with poorer outcomes, its prognostic significance may differ in the context of immune checkpoint blockade. In AFP-high tumors, the potential for reinvigorating intratumoral T-cell exhaustion may determine therapeutic response. Future studies linking AFP, immunophenotypes, and clinical outcomes are needed to clarify these relationships.

## Supplementary Information

Below is the link to the electronic supplementary material.

Supplementary Material 1

## Data Availability

The data supporting the findings of this study are available within the paper and its Supplementary Information, and more details are available from the authors.

## References

[1] R.L. Siegel, K.D. Miller, N.S. Wagle, A. Jemal, CA: Cancer J. Clin. **73**, 17–48 (2023) 10.3322/caac.21763 10.3322/caac.21763 36633525

[2] S.L. Chan, H.C. Sun, Y. Xu, H. Zeng, H.B. El-Serag, J.M. Lee, M.E. Schwartz, R.S. Finn, J. Seong, X.W. Wang, V. Paradis, G.K. Abou-Alfa, L. Rimassa, J.H. Kao, B.H. Zhang, J.M. Llovet, J. Bruix, T.C. Yip, V.W. Wong, G.L. Wong, L.L. Chan, M.Q. Liu, Q. Gao, F. Shen, R.K. Kelley, A.L. Cheng, M. Kurosaki, H. Toyoda, W.X. Chen, T. Murakami, P. Liang, J. Zucman-Rossi, Y. Minami, S. Miyayama, K. Wang, K. Man, K. Hasegawa, Q. Li, K. Tsuchiya, L. Xu, V. Chew, P. Chow, Y. Hoshida, A. Lujambio, I.O. Ng, M. Sakamoto, Y.N. Park, T. Yau, M. Kudo, J. Fan, J. Zhou, Lancet (London England). **406**, 731–778 (2025). 10.1016/s0140-6736(25)01042-6 40744051 10.1016/S0140-6736(25)01042-6

[3] Z. Macek Jilkova, J. Ghelfi, T. Decaens, Curr. Opin. Oncol. **34**, 155–160 (2022). 10.1097/cco.0000000000000812 34923550 10.1097/CCO.0000000000000812

[4] P.R. Galle, F. Foerster, M. Kudo, S.L. Chan, J.M. Llovet, S. Qin, W.R. Schelman, S. Chintharlapalli, P.B. Abada, M. Sherman, A.X. Zhu, Liver Int. **39**, 2214–2229 (2019). 10.1111/liv.14223 31436873 10.1111/liv.14223

[5] Y. Xu, Q. Guo, L. Wei, Cancers. **13**, 5096 (2021) 34680245 10.3390/cancers13205096 PMC8534193

[6] M. Biselli, F. Conti, A. Gramenzi, M. Frigerio, A. Cucchetti, G. Fatti, M. D’Angelo, M. Dall’Agata, E.G. Giannini, F. Farinati, F. Ciccarese, P. Andreone, M. Bernardi, F. Trevisani, Br. J. Cancer. **112**, 69–76 (2015). 10.1038/bjc.2014.536 25314061 10.1038/bjc.2014.536 PMC4453600

[7] S.L. Chan, F. Mo, P.J. Johnson, D.Y. Siu, M.H. Chan, W.Y. Lau, P.B. Lai, C.W. Lam, W. Yeo, S.C. Yu, HPB: Official J. Int. Hepato Pancreato Biliary Association. **16**, 366–372 (2014). 10.1111/hpb.12146 10.1111/hpb.12146 PMC3967889 23980880

[8] S.I. Malov, I.V. Malov, A.G. Kuvshinov, P.N. Marche, T. Decaens, Z. Macek-Jilkova, N.D. Yushchuk, Sovrem Tekhnologii Med. **13**, 27–33 (2021). 10.17691/stm2021.13.1.03 34513063 10.17691/stm2021.13.1.03 PMC8353694

[9] P.V. Munson, J. Adamik, L.H. Butterfield, Trends Immunol. **43**, 438–448 (2022). 10.1016/j.it.2022.04.001 35550875 10.1016/j.it.2022.04.001

[10] A. Gurakar, M. Ma, J. Garonzik-Wang, A. Kim, R.A. Anders, K. Oshima, C. Georgiades, M. Gurakar, S. Ottmann, A.M. Cameron, B. Philosophe, B. Saberi, Ann. Hepatol. **17**, 1052–1066 (2018). 10.5604/01.3001.0012.7206 31208632 10.5604/01.3001.0012.7206

[11] X. Hu, R. Chen, Q. Wei, X. Xu, Int. J. Biol. Sci. **18**, 536–551 (2022). 10.7150/ijbs.64537 35002508 10.7150/ijbs.64537 PMC8741863

[12] Q.T. Li, M.J. Qiu, S.L. Yang, X. Fang, X.X. He, M.M. Wang, Y.N. Li, Z.F. Xiong, S. Huang, J. Oncol. 2020, 9327512 (2020) 10.1155/2020/9327512 10.1155/2020/9327512 PMC7407027 32774373

[13] C. Esteban, P. Terrier, C. Frayssinet, J. Uriel, Tumor Biology. **17**, 299–305 (2009). 10.1159/000217992 10.1159/000217992 8792856

[14] B. Lin, Q. Wang, K. Liu, X. Dong, M. Zhu, M. Li, Front. Oncol. **11** (2021). 10.3389/fonc.2021.625936 10.3389/fonc.2021.625936 PMC7947232 33718192

[15] Z.M. Jilkova, C. Aspord, K. Kurma, A. Granon, C. Sengel, N. Sturm, P.N. Marche, T. Decaens, Clin. Translational Gastroenterol. **10**, (2019) 10.14309/ctg.0000000000000058 PMC6708670 31295151

[16] L. Sun, G. Xu, W. Liao, H. Yang, H. Xu, S. Du, H. Zhao, X. Lu, X. Sang, Y. Mao, Oncotarget. **8**, 39658–39672 (2017). 10.18632/oncotarget.17340 28487498 10.18632/oncotarget.17340 PMC5503641

[17] H. He, S. Chen, Z. Fan, Y. Dong, Y. Wang, S. Li, X. Sun, Y. Song, J. Yang, Q. Cao, J. Jiang, X. Wang, W. Wen, H. Wang, Cell. Discovery. **9**, 60 (2023). 10.1038/s41421-023-00563-x 37336873 10.1038/s41421-023-00563-x PMC10279759

[18] Z. Ge, G. Zhou, L. Campos Carrascosa, E. Gausvik, P.P.C. Boor, L. Noordam, M. Doukas, W.G. Polak, T. Terkivatan, Q. Pan, R.B. Takkenberg, J. Verheij, J.I. Erdmann, I.J. JNM, M.P. Peppelenbosch, J. Kraan, J. Kwekkeboom, D. Sprengers, Cell. Mol. Gastroenterol. Hepatol. **12**, 443–464 (2021). 10.1016/j.jcmgh.2021.03.003 33781741 10.1016/j.jcmgh.2021.03.003 PMC8255944

[19] H.D. Kim, G.W. Song, S. Park, M.K. Jung, M.H. Kim, H.J. Kang, C. Yoo, K. Yi, K.H. Kim, S. Eo, D.B. Moon, S.M. Hong, Y.S. Ju, E.C. Shin, S. Hwang, S.H. Park, Gastroenterology, **155**, 1936–1950.e1917 (2018) 10.1053/j.gastro.2018.08.030 10.1053/j.gastro.2018.08.030 30145359

