# Supplementary material for: Intratumoral PD-1high CD8+ T cells correlate with AFP levels in HCC patients: a brief report
Source: Cell Oncol (Dordr). 2026 Feb 3;49(1):39. doi: 10.1007/s13402-026-01170-0 (PMC12868040; doi:10.1007/s13402-026-01170-0)
Supplement: Supplementary file 1 — Supplementary Material 1 [file 13402_2026_1170_MOESM1_ESM.docx]

**Supplementary Information:**

**Intratumoral PD-1^+^CD8^+^ T cells correlate with AFP levels in HCC patients: a brief report**

Macek Jilkova Zuzana^1,2^, Ghelfi Julien^1,3^, Dumolard Lucile^1^, Sengel Christian^3^, Brusset Bleuenn^2^, Teyssier Yann^3^, Costentin Charlotte^1,2^, Decaens Thomas^1,2^

^1^ Univ. Grenoble Alpes, France; Institute for Advanced Biosciences, Research Center UGA / Inserm U 1209 / CNRS 5309, Grenoble, France

^2^ Hepato-gastroenterology and digestive oncology department, CHU Grenoble Alpes, Grenoble France

^3^ Service de radiologie, CHU Grenoble Alpes, France

**MATERIAL AND METHODS**

**Patients and sample processing**

93 patients suffering from hepatocellular carcinoma (HCC) were included in this study and were selected prior to treatment (Hepato-gastroenterology and digestive oncology department, CHU Grenoble-Alpes). Exclusion criteria were no immunophenotyping at the time of sampling performed and no pathologic evidence of HCC in tumor biopsy. Fresh blood samples were collected for multiparametric flow cytometry before the core liver biopsy procedure. Liver biopsies (tumor and non-tumor tissues) were collected and divided into two parts. One part was used for histological examination assessed by experienced liver pathologists, to confirm HCC diagnosis and define whether biopsy was performed within HCC or not, whereas the other part was processed within hours (≤ 4h) following the clinical biopsy to conduct extensive phenotypic immunological analyses on fresh samples.

Additional samples from five alpha-fetoprotein (AFP)-negative early stage HCC patients were included for *ex-vivo* analysis. This study was performed in accordance with the Declaration of Helsinki and the French legislation based on local sample collection (DC-2014-2295 and AC-2019-3627), and all of its participants provided written informed consent.

**Flow cytometry analyses – Immunophenotyping of HCC patients**

Immediately after the liver biopsy, tumor and non-tumor samples were transferred in the Hypothermosol™ FRS solution to research laboratory. Cells were recovered through mechanical disruption. Blood and intrahepatic cell suspensions were divided into two tubes and immunostained without any stimulation with the following anti-human antibodies of surface markers: anti-CD45-APC/Cy7 (clone HI30, BioLegend), anti-CD3-PerCP-Cy5.5 (clone UCHT1, BioLegend), anti-CD56-BV605 (clone HCD56, BioLegend), anti-CD16-AF700 (clone 3G8, BioLegend), anti-CD8-PE/Cy7 (clone RPA-T8, BD Biosciences), anti-CD69-PE (clone FN50, BioLegend), anti-CTLA4-BV421 (clone BNI3, BioLegend), anti-PD-1-BV711 (clone EH12.2H7, BioLegend), anti-LAG3-PE (clone 3DS223H, eBioscience), anti-OX40-FITC (clone Ber-ACT35, BioLegend), anti-4-1BB-BV421 (clone 4B4-1, BioLegend), and anti-TIM3-BV785 (clone F39-2E2, BioLegend). FluoroFix™ Buffer (BioLegend) was used to fix cells. The Zombie UV™ Fixable Viability kit was used to exclude dead cells, and Fluorescence Minus One (FMO) controls were used to determine positive immune cell population. Data were acquired with the BD-LSRII flow cytometer (BD Biosciences), collected using BD FACSDiva 6.3.1 software and analyzed using FCS Express 7 Flow software, using the previously described gating strategy [1, 2]. In order to obtain a sufficiently powered and clinically representative patient cohort, analyses of fresh samples were performed over a period of two years. Each experiment was conducted using experiment-specific compensation matrices and FMO controls to ensure accurate identification of positive populations. Data are presented as immune cell population frequencies.

**Cytokine production by T cells following exposure to AFP peptides**

Peripheral blood mononuclear cells (PBMCs) and cell suspensions obtained by enzymatic digestion from tumoral and non-tumoral liver biopsies of five hepatocellular carcinoma (HCC) alpha-fetoprotein (AFP) negative patients were cryopreserved in liquid nitrogen. Samples were thawed, washed, and plated at 2 × 10⁵ cells per well in 96-well plates. Cells were treated with either dimethyl sulfoxide (DMSO) as a control or an AFP peptide pool dissolved in DMSO at final concentration of 2 µg/mL, (PepMix Human AFP, 150 peps; JPT Peptide Technologies GmbH, Germany). Cells were then stimulated with Phorbol 12-myristate-13-acetate and ionomycin activation cocktail with Brefeldin A (BioLegend) to allow intracellular cytokine accumulation. Cells were incubated for 5 h at 37°C in a humidified atmosphere with 5% CO₂. Following incubation, cells were stained for flow cytometric analysis. Cell viability was assessed using Zombie Green viability dye. Surface staining was performed using the following antibodies: anti-CD45-APC/Cy7 (clone HI30, BioLegend), anti-CD3-PerCP-Cy5.5 (clone UCHT1, BioLegend), anti-CD56-APC (clone HCD56, BioLegend), anti-CD19-APC (clone 2H7, BioLegend), anti-CD8-PE/Cy7 (clone RPA-T8, BioLegend), anti-CD4-BV510 (clone SK3, BioLegend), and anti-PD-1-BV711 (clone EH12.2H7, BioLegend). For intracellular cytokine detection, cells were fixed and permeabilized using the intracellular fixation and permeabilization buffer set (Thermo Fisher Scientific) and stained with antibodies against IFN-γ-PE (clone B27, BD Pharmingen) and IL-10 (clone JES3-9D7, BioLegend). Data were acquired on a flow cytometer and analyzed by FCS Express 7 Flow software using a sequential gating strategy to identify T cell populations. Initially, doublets were excluded based on forward scatter height versus forward scatter area (FSC-H vs. FSC-A), lymphocytes were then identified according to their characteristic forward and side scatter properties (FSC-A vs. SSC-A). From this population, live cells were selected using a viability dye, followed by gating on CD45⁺ leukocytes. To remove non–T cell lineages, CD19⁺ B cells and CD56⁺ natural killer cells, both detected in the APC channel, were excluded. The remaining cells were gated on CD3 expression (PE-Cy5.5) to define total T cells. Finally, CD3⁺ T cells were subdivided into CD4⁺ T cells (BV510) and CD8⁺ T cells (PE-Cy7) for downstream analyses.

**Statistical analysis**

Standard descriptive statistics were used for continuous quantitative variables, presented as medians and interquartile ranges. Analyses were performed using the statistical software GraphPad Prism 10.6.1 (GraphPad Software). Normal distribution was tested by means of the D’Agostino–Pearson omnibus normality test. When data from either cohort were not normally distributed, the Kruskal-Wallis test was used to compare three groups (Tumor, Non-Tumor, Blood), and the Mann–Whitney test was performed to compare two groups (AFP<10 vs AFP≥10 groups). The Spearman rank correlation was used to examinate the relationship between two variables (non-parametric). A p value of < 0.05 was considered significant.


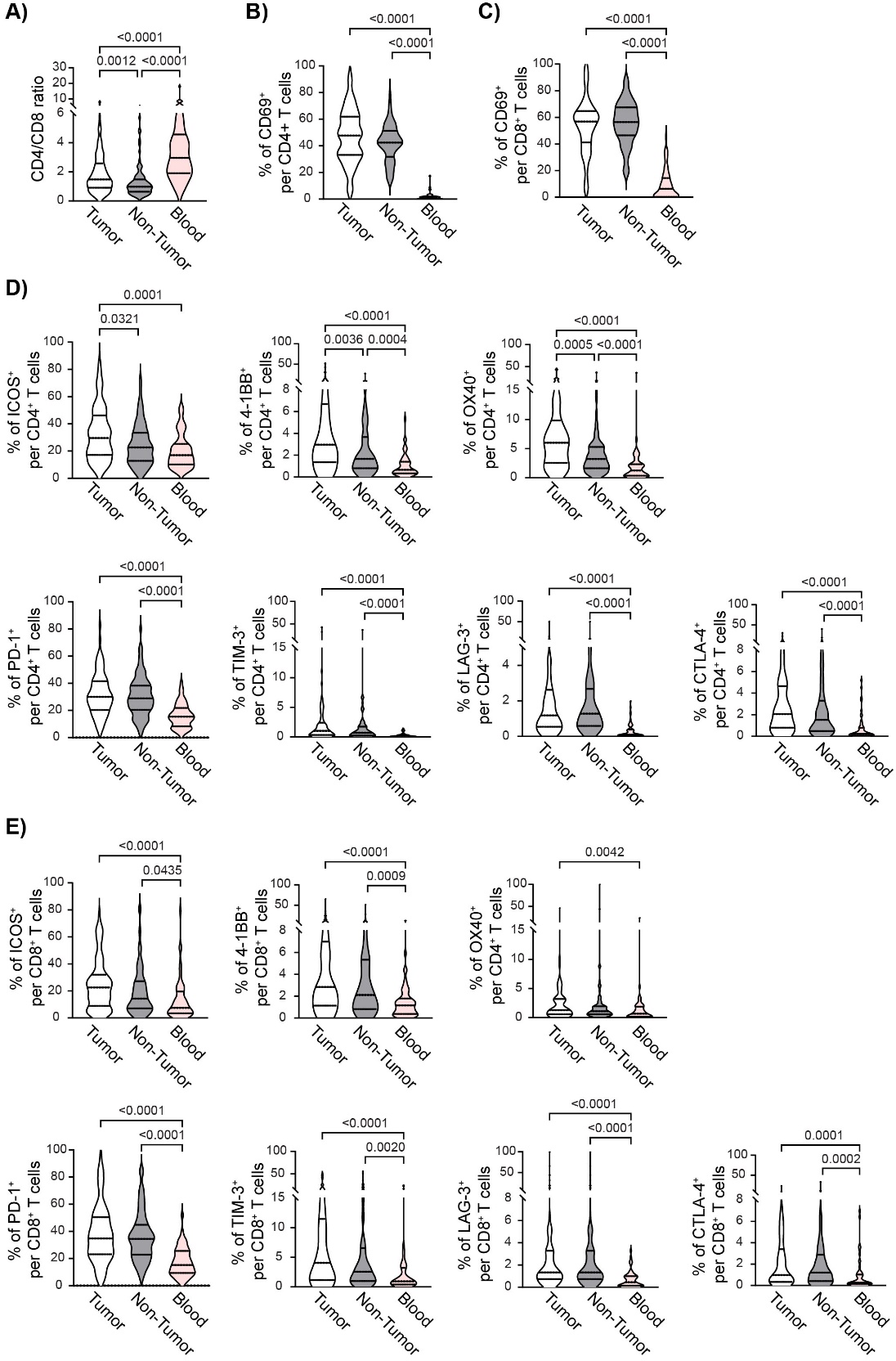


**Figure S1: Immunophenotyping of HCC patients.** A) CD4/CD8 ratio in Tumor, Non-Tumor and Blood samples. B) The frequency of activated CD69^+^ cells per CD4^+^T cell population. C) The frequency of activated CD69^+^ cells per CD8^+^T cell population. D) The frequency of ICOS^+^, 4-1BB^+^, OX40^+^, PD-1^+^, TIM-3^+^, LAG-3^+^ and CTLA-4^+^ cells per CD4^+^T cell population in Tumor, Non-Tumor and Blood samples. E) The frequency of ICOS^+^, 4-1BB^+^, OX40^+^, PD-1^+^, TIM-3^+^, LAG-3^+^ and CTLA-4^+^ cells per CD8^+^T cell population in Tumor, Non-Tumor and Blood samples. Groups were compared by Kruskal-Wallis test, (n=93).


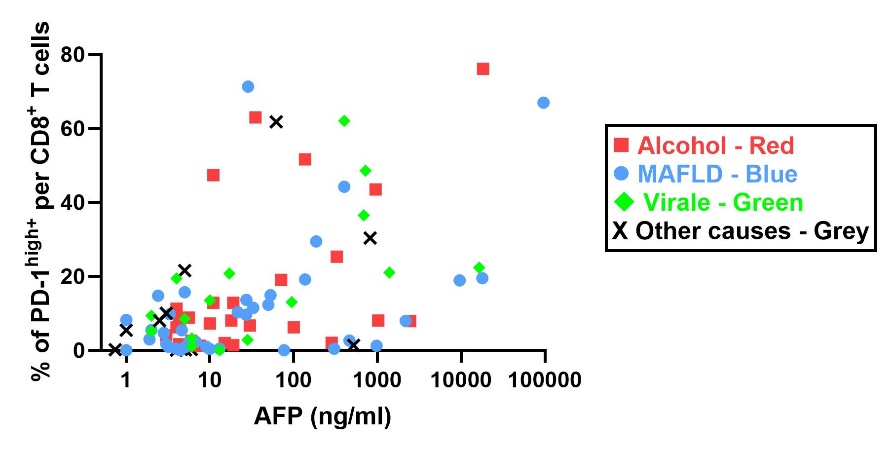


| **Figure S2: Correlation between intra-tumoral PD-1^high^CD8^+^ T cell frequency and AFP circulating levels in HCC patients,** **stratified by liver disease etiology.** Each dot represents one patient (n = 93). Colors indicate HCC etiology: Alcohol – red (n=28); Metabolic dysfunction–associated steatohepatitis (MASH) – blue (n=37); Viral hepatitis – green (n=16); Other causes – grey (n=12).   \| 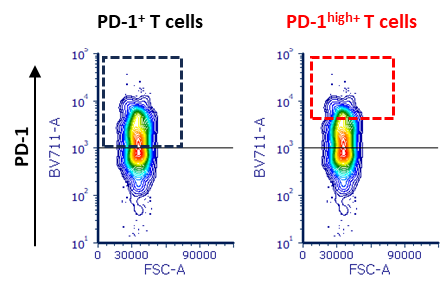 \| \| --- \| \| **Figure S3: Representative flow cytometry plots illustrating PD-1 expression on intratumoral T cells.** PD-1 was detected using BV711, and gates were set to discriminate positive (PD-1^+^ T cells) and high (PD-1^high+^ T cells) T-cell populations. \|  \| 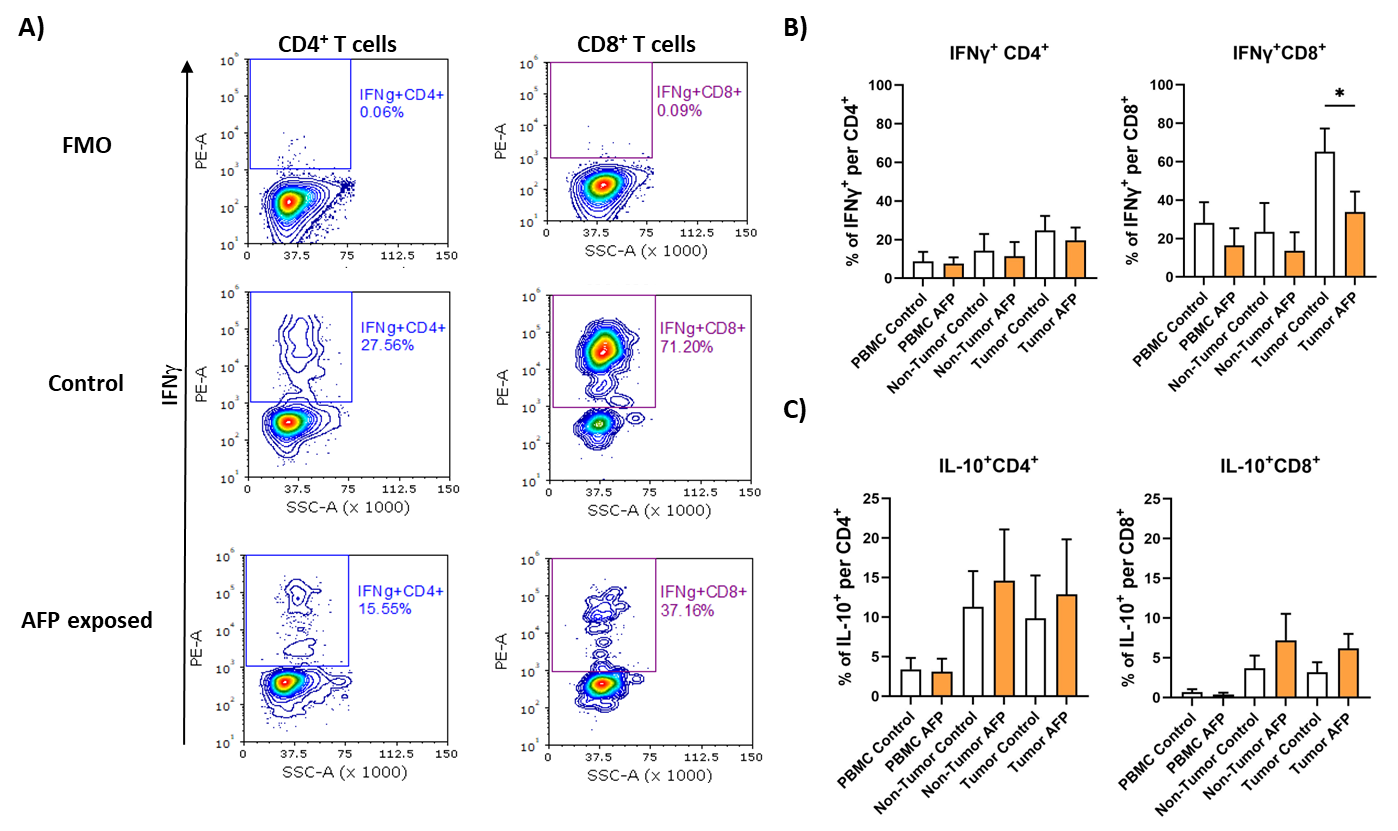 \| \| --- \| \| **Figure S4: Cytokine production by T cells following exposure to AFP peptides.** Multiparametric flow cytometry was used to assess the functionality of T cells after the exposure to AFP peptides in AFP-negative HCC patients (to avoid potential confounding effects of AFP-specific re-challenge). (A) Representative graphs of IFNγ production by CD4^+^ T cells and CD8^+^ T cells, stimulated for 5 hours with a Phorbol 12-myristate-13-acetate and ionomycin activation cocktail with Brefeldin A. Fluorescence Minus One controls (FMO) were used to determine positive immune cell population. Cells were treated with dimethyl sulfoxide (DMSO) as a control or with an AFP peptide pool prepared in DMSO and applied at a final concentration of 2 µg/mL. (B) Frequencies of IFNγ-expressing CD4⁺ T cells and CD8⁺ T cells. (C) Frequencies of IL-10-expressing CD4⁺ T cells and CD8⁺ T cells. Statistical comparisons were performed using the Wilcoxon pair test for Control vs AFP-exposed conditions: *p-value < 0.05, n=5. AFP peptide pool exposed conditions are represented in orange. \| |
| --- | --- | --- | --- | --- |

**SI Table 1. Frequency of immune cell populations of HCC patients.** Kruskal-Wallis test was used to compare Tumor, Non-Tumor and Blood immunophenotyping data, (n=93). **** p<0.0001, compared to Blood; *** p<0.001, compared to Blood; ** p<0.01, compared to Blood; * p<0.05, compared to Blood; ^###^ p<0.001, compared to Non-Tumor; ^##^ p<0.01, compared to Non-Tumor; ^#^ p<0.05, compared to Non-Tumor.

|  | **Tumor** | **Non-Tumor** | **Blood** | Kruskal-Wallis test |
| --- | --- | --- | --- | --- |
|  |  |  |  |  |
| CD69^+^ T cells (%), median [IQR] | 51.04****  [37.28-63.28] | 48.87****  [40.20-59.43] | 2.39  [0.16-5.33] | <0.0001 |
| ICOS^+^ T cells (%), median [IQR] | 26.65***^, ##^  [13.79-40.42] | 17.75  [10.10-29.79] | 14.42  [8.29-22.72] | <0.0001 |
| 4-1BB^+^ T cells (%), median [IQR] | 3.62****^, ##^  [1.78-7.72] | 1.97****  [0.91-4.15] | 0.83  [0.32-1.53] | <0.0001 |
| OX40^+^ T cells (%), median [IQR] | 4.21****^, ###^  [1.66-6.87] | 2.39**  [1.10-3.40] | 1.19  [0.33-2.22] | <0.0001 |
| PD-1^+^ T cells (%), median [IQR] | 32.79****  [24.86-45.57] | 33.00****  [25.16-41.76] | 15.54  [8.81-22.75] | <0.0001 |
| TIM-3^+^ T cells (%), median [IQR] | 1.89****  [0.75-7.15] | 1.72****  [0.69-4.04] | 0.36  [0.20-1.15] | <0.0001 |
| LAG-3^+^ T cells (%), median [IQR] | 1.81****  [0.67-3.41] | 1.32****  [0.67-2.92] | 0.19  [0.08-0.51] | <0.0001 |
| CTLA-4^+^ T cells (%), median [IQR] | 1.82****  [0.60-4.36] | 1.39****  [0.57-3.42] | 0.26  [0.11-0.92] | <0.0001 |
|  |  |  |  |  |
| CD69^+^ NKT cells (%), median [IQR] | 71.32****  [37.28-63.28] | 69.62****  [40.20-59.43] | 15.64  [0.16-5.33] | <0.0001 |
| ICOS^+^ NKT cells (%), median [IQR] | 22.73****^, #^  [11.33-36.45] | 14.85**  [9.23-25.83] | 7.65  [3.45-16.24] | <0.0001 |
| 4-1BB^+^ NKT cells (%), median [IQR] | 7.47****  [3.24-14.10] | 5.19****  [2.92-10.02] | 2.15  [1.07-4.51] | <0.0001 |
| OX40^+^ NKT cells (%), median [IQR] | 13.16****  [6.02-26.53] | 9.38****  [4.55-20.13] | 1.82  [0.83-6.53] | <0.0001 |
| PD-1^+^ NKT cells (%), median [IQR] | 20.67***  [11.58-32.78] | 22.01***  [13.29-34.90] | 12.06  [6.36-22.92] | 0.0001 |
| TIM-3^+^ NKT cells (%), median [IQR] | 7.00****  [3.40-13.51] | 6.55****  [2.74-11.90] | 2.64  [1.35-5.49] | <0.0001 |
| LAG-3^+^ NKT cells (%), median [IQR] | 17.98****  [6.93-37.14] | 11.74****  [5.30-28.17] | 1.74  [0.49-4.75] | <0.0001 |
| CTLA-4^+^ NKT cells (%), median [IQR] | 5.58****  [2.31-10.70] | 4.77****  [1.89-11.61] | 1.94  [0.88-3.84] | <0.0001 |
|  |  |  |  |  |
| CD69^+^ NK cells (%), median [IQR] | 46.86****^, ##^  [32.39-57.39] | 57.72****  [47.51-69.00] | 7.81  [0.62-15.07] | <0.0001 |
| ICOS^+^ NK cells (%), median [IQR] | 1.69***  [0.79-4.76] | 1.43*  [0.60-3.39] | 0.90  [0.25-1.58] | 0.0005 |
| 4-1BB^+^ NK cells (%), median [IQR] | 2.78***  [1.19-6.91] | 2.34*  [1.04-4.61] | 1.30  [0.84-2.51] | 0.0012 |
| OX40^+^ NK cells (%), median [IQR] | 4.80***  [1.63-9.31] | 3.25  [1.56-6.02] | 2.05  [0.70-3.89] | 0.0004 |
| PD-1^+^ NK cells (%), median [IQR] | 1.76  [0.61-3.77] | 1.67  [0.57-4.32] | 1.28  [0.39-2.46] | 0.1804 |
| TIM-3^+^ NK cells (%), median [IQR] | 5.64****  [2.61-8.35] | 4.24**  [2.21-7.59] | 2.09  [1.16-4.02] | <0.0001 |
| LAG-3^+^ NK cells (%), median [IQR] | 3.04****  [1.09-7.04] | 2.29***  [1.18-5.44] | 0.77  [0.27-2.12] | <0.0001 |
| CTLA-4^+^ NK cells (%), median [IQR] | 3.95****  [1.41-8.73] | 3.29****  [1.52-6.83] | 1.01  [0.40-2.85] | <0.0001 |

**SI Table 2. Frequency of ICM+ T cells in the tumor of HCC patients with circulating AFP levels lower than 10 ng/ml (AFP<10) compared to tumor of HCC patients with circulating AFP levels equal or higher than 10 ng/ml (AFP≥10).** Mann-Whitney U test was used to compare data of AFP<10 and AFP≥10 group of patients, 2-tailed P value.

|  | AFP < 10  (n=43) | AFP ≥ 10  (n=50) | *p* value |
| --- | --- | --- | --- |
| Tumor : T cells |  |  |  |
| CD69^+^ T cells (%), median [IQR] | 50.74 [34.46-60.20] | 54.08 [38.96-65.68] | 0.2683 |
| ICOS^+^ T cells (%), median [IQR] | 24.18 [11.07-35.09] | 29.65 [17.54-47.35] | 0.1088 |
| 4-1BB^+^ T cells (%), median [IQR] | 2.78 [1.81-5.58] | 4.09 [1.65-9.69] | 0.1299 |
| OX40^+^ T cells (%), median [IQR] | 4.10 [1.39-6.62] | 4.37 [2.77-8.02] | 0.5007 |
| PD-1^+^ T cells (%), median [IQR] | 29.30 [19.19-38.63] | 37.26 [26.68-54.16] | 0.0044 |
| PD-1^high^ T cells (%), median [IQR] | 4.09 [1.08-8.49] | 9.71 [4.24-23.59] | 0.0004 |
| TIM-3^+^ T cells (%), median [IQR] | 1.71 [0.76-3.81] | 3.45 [0.73-8.49] | 0.1752 |
| LAG-3^+^ T cells (%), median [IQR] | 1.19 [0.56-2.74] | 1.84 [0.70-3.80] | 0.2830 |
| CTLA-4^+^ T cells (%), median [IQR] | 1.73 [0.38-4.26] | 1.88 [0.87-4.59] | 0.5330 |
|  |  |  |  |
| Tumor : CD4^+^ T cells |  |  |  |
| CD69^+^ CD4^+^ T cells (%), median [IQR] | 50.24 [33.61-60.61] | 59.21 [50.02-67.92] | 0.9583 |
| ICOS^+^ CD4^+^ T cells (%), median [IQR] | 25.98 [16.09-40.98] | 33.07 [19.90-51.43] | 0.1304 |
| 4-1BB^+^ CD4^+^ T cells (%), median [IQR] | 2.67 [1.37-4.20] | 3.40 [1.61-8.97] | 0.2661 |
| OX40^+^ CD4^+^ T cells (%), median [IQR] | 6.04 [2.22-8.90] | 5.94 [3.29-10.97] | 0.5742 |
| PD-1^+^ CD4^+^ T cells (%), median [IQR] | 26.13 [18.51-36.74] | 31.48 [24.38-45.98] | 0.0236 |
| PD-1^high^ CD4^+^ T cells (%), median [IQR] | 4.64 [1.04-7.81] | 7.81 [3.40-13.42] | 0.0224 |
| TIM-3^+^ CD4^+^ T cells (%), median [IQR] | 0.78 [0.29-1.60] | 1.40 [0.36-4.24] | 0.0549 |
| LAG-3^+^ CD4^+^ T cells (%), median [IQR] | 1.18 [0.34-2.34] | 1.13 [0.60-2.80] | 0.3825 |
| CTLA-4^+^ CD4^+^ T cells (%), median [IQR] | 2.32 [0.47-4.08] | 2.00 [0.84-4.87] | 0.4371 |
|  |  |  |  |
| Tumor : CD8^+^ T cells |  |  |  |
| CD69^+^ CD8^+^ T cells (%), median [IQR] | 50.24 [33.61-60.61] | 59.21 [50.02-67.92] | 0.0137 |
| ICOS^+^ CD8^+^ T cells (%), median [IQR] | 19.08 [5.70-27.88] | 24.90 [13.15-37.01] | 0.0985 |
| 4-1BB^+^ CD8^+^ T cells (%), median [IQR] | 2.60 [1.20-5.33] | 3.39 [1.08-9.75] | 0.2761 |
| OX40^+^ CD8^+^ T cells (%), median [IQR] | 1.42 [0.52-3.19] | 1.29 [0.57-3.25] | 0.9343 |
| PD-1^+^ CD8^+^ T cells (%), median [IQR] | 28.82 [16.49-43.05] | 39.97 [27.47-57.00] | 0.0007 |
| PD-1^high^ CD8^+^ T cells (%), median [IQR] | 3.63 [0.96-8.50] | 13.03 [6.47-29.92] | <0.0001 |
| TIM-3^+^ CD8^+^ T cells (%), median [IQR] | 2.66 [1.14-8.75] | 5.65 [0.99-13.47] | 0.1396 |
| LAG-3^+^ CD8^+^ T cells (%), median [IQR] | 1.26 [0.77-3.26] | 1.90 [0.67-5.74] | 0.4059 |
| CTLA-4^+^ CD8^+^ T cells (%), median [IQR] | 0.94 [0.32-3.50] | 1.17 [0.43-3.37] | 0.5130 |

**References:**

1 Z. Macek Jilkova, M.N. Hilleret, T. Gerster, N. Sturm, M. Mercey-Ressejac, J.-P. Zarski, V. Leroy, P.N. Marche, C. Costentin and T. Decaens, Cells 10, 2671 (2021)

2 Z. Macek Jilkova, C. Aspord, K. Kurma, A. Granon, C. Sengel, N. Sturm, P.N. Marche and T. Decaens, Clinical and translational gastroenterology 10, (2019)
